# Experiences and Perceptions of Telephone-delivery of the Common Elements Treatment Approach for Mental Health Needs Among Young People in Zambia During the COVID-19 Pandemic

**DOI:** 10.3389/fpubh.2022.906509

**Published:** 2022-10-13

**Authors:** Saphira Munthali-Mulemba, Caleb J. Figge, Kristina Metz, Jeremy C. Kane, Stephanie Skavenski, Mwamba Mwenge, Brandon A. Kohrt, Gloria A. Pedersen, Izukanji Sikazwe, Laura K. Murray

**Affiliations:** ^1^Centre for Infectious Disease Research in Zambia (CIDRZ), Lusaka, Zambia; ^2^Department of Mental Health, Johns Hopkins Bloomberg School of Public Health, Baltimore, MD, United States; ^3^Department of Epidemiology, Columbia University Mailman School of Public Health, New York, NY, United States; ^4^Department of Psychiatry, George Washington University School of Medicine and Health Sciences, Washington, DC, United States

**Keywords:** mental health, telehealth, CETA, COVID-19, adolescents and young adults

## Abstract

**Background:**

Mental and behavioral health needs are immense in low-to-middle income countries (LMIC), particularly for adolescents and young adults (AYA). However, access to mental health services is limited in LMIC due to barriers such as distance to a health care site, low number of providers, and other structural and logistical challenges. During the COVID-19 pandemic, these barriers were significantly exacerbated and, thus, mental health services were severely disrupted. A potential solution to some of these barriers is remote delivery of such services via technology. Exploration of AYA experiences is needed to understand the benefits and challenges when shifting to remotely delivered services.

**Methods:**

Participants included 16 AYA (15–29 years) residing in Lusaka, Zambia who met criteria for a mental or behavioral health concern and received telehealth delivery of the Common Elements Treatment Approach (CETA). AYA participated in semi-structured qualitative interviews to explore feasibility, acceptability, and barriers to telephone-delivered treatment in this context. Thematic coding analysis was conducted to identify key themes.

**Findings:**

Three major response themes emerged: 1) Advantages of telehealth delivery of CETA, Disadvantages or barriers to telehealth delivery of CETA, 3) AYA recommendations for optimizing telehealth (ways to improve telehealth delivery in Zambia. Results indicate that logistical and sociocultural barriers i.e., providing AYA with phones to use for sessions, facilitating one face-to-face meeting with providers) need to be addressed for success of remotely delivered services.

**Conclusion:**

AYA in this sample reported telehealth delivery reduces some access barriers to engaging in mental health care provision in Zambia. Addressing logistical and sociocultural challenges identified in this study will optimize feasibility of telehealth delivery and will support the integration of virtual mental health services in the Zambian health system.

## Introduction

Mental and behavioral health needs among adolescents and young adults (AYA) are high (1, 2), with mental and behavioral health problems accounting for up to 30% of disability- adjusted life years before the age of 30. In Zambia, rates of psychosocial distress among AYA are significant, with prevalence estimates over 15% for adolescents in Zambia (2). High levels of poverty in the country are noted as a major catalyst for mental health problems, especially for unemployed youth and people with disabilities (2). Over 43% of Zambian AYA are living in poverty, and most recent estimates in 2022 of the general population show unemployment rate of 13.81%, according to the world bank collection of development indicators.

Economic conditions for Zambians were worsened by the COVID-19 pandemic, exacerbating unemployment, economic inequality, and disparities in access to healthcare services. These stressors are known to be causally associated with a range of poor mental and behavioral health outcomes for AYA in LMIC, including emotional and/or behavioral dysregulation, aggression, risky sexual behaviors, substance abuse, and difficulties with functioning (3).

The quality of available mental and behavioral health supports is often deficient in LMIC, particularly for AYA (4). In Zambia, <1% of the national health budget is dedicated to mental health and limited trained providers. Logistical barriers, such as distance to a health care site and low number of providers (5) met with high rates of mental and behavioral concerns further contribute to a substantial treatment gap in Zambia. These barriers were exacerbated during the COVID-19 pandemic when in-person mental health services were severely disrupted (5).

One approach with considerable evidence in expanding access to evidence-based training and treatment in LMIC is the Common Elements Treatment Approach (CETA). CETA is designed for delivery by a range of providers, including nonprofessional providers without formal mental and behavioral health education or training. Providers are trained in clinical decision making to effectively treat multiple comorbid problems at once, including trauma, depression, anxiety, violence, substance use, safety, relationship problems, and behavioral problems. CETA is the only transdiagnostic intervention for LMIC that is multi-problem (i.e., addressing many different problem areas) and flexible (i.e., does not need to have a set number of sessions, or the same elements for everyone) (6–10). Randomized controlled trials (RCT) have demonstrated the effectiveness of CETA for a range of mental health, substance use, and behavioral issues in a variety of low-resource settings, including in Zambia (10). During the COVID-19 pandemic, a telehealth delivery option for CETA (T-CETA) was developed for piloting and testing in Zambia as part of the World Health Organization (WHO) Ensuring Quality in Psychosocial Support (EQUIP) project consortium, in partnership with the George Washington University.

The WHO EQUIP platform is a digital resource including competency assessment tools and guidance on how to rate competency, provide competency-based feedback, and conduct competency-driven training (11).

Telehealth service delivery *via* telephone has been a widespread approach globally for increasing access and maintaining healthcare provision during the COVID-19 pandemic in efforts to reduce logistical barriers to accessing a healthcare facility and to maintain safety guidelines. In Zambia, synchronous telephone delivery has proven most feasible, compared to asynchronous and video connections, particularly for common mental health problems such as anxiety and depression (12).

Even prior to COVID-19, the use of telehealth methods was well reviewed and highlighted its utility in LMIC contexts to increasing access to care (13). Given the rapid expansion of access to smartphones in Zambia (14), the population is well-positioned to utilize technology for receiving care. Although telehealth strategies are proven effective in some LMIC (15, 16), it's critical to understand contextual dynamics that impact the uptake of telehealth methods in each specific population. The sociocultural feasibility, acceptability, and sustainability of telehealth delivery is unknown in Zambia. Exploration of telehealth experiences is needed to understand the benefits and challenges when shifting to remotely delivered services, especially for high-risk groups, such as AYA.

The current study explored AYA experiences, perceptions, and recommendations for synchronous telephone delivery of an evidence-based mental and behavioral health intervention in Zambia. A better understanding of the advantages and barriers to telehealth in the Zambian context can inform intervention and programming development to optimize access and quality of mental health service delivery. Our research aims were to identify:

1. Perceived advantages of receiving mental/behavioral health care via telehealth.
2. Real and perceived barriers to participating in telehealth care services.
3. Recommendations for optimizing telehealth delivery in the Zambian context.

## Materials and Methods

### Participants and Recruitment

Adolescent and young adult (AYA) participants were randomly selected to participate from a larger T-CETA pilot study in Lusaka, Zambia. The pilot study included 25 participants ranging in age from 15 to 29 who reported experiences of mental or behavioral health problems, traumatic experiences, substance use, physical or sexual violence, or stress related to COVID-19. Participants were recruited through a range of service delivery settings in Lusaka including primary health clinics, hospital/university settings, and community care sites, leveraging multiple service delivery systems (e.g., HIV care, maternal care, education, social and religious settings). Sixteen pilot participants (8 females and 8 males) were randomly selected to participate in the qualitative interviews and are included in the present analysis. All participants provided written consent prior to the commencement of data collection. Parental permission and assent were also obtained for AYA under age 18. The Johns Hopkins Bloomberg School of Public Health Institutional Review Board, the University of Zambia Biomedical Research Ethics Committee, the George Washington University (GWU) Institutional Review Board Office, approval number—IRB# NCR191797, and the World Health Organization (WHO) Research Ethics Review Committee—ERC.0003192 approved this study.

### T-CETA Intervention

A telephone-delivered version of CETA (T-CETA) was developed in response to the COVID-19 pandemic and associated public health guidelines and safety restrictions. The CETA manual was adapted for T-CETA following a review of evidence-based telehealth strategies, ethical guidelines, and clinical recommendations from telehealth providers. Additions to the manual included telehealth-specific discussions of confidentiality and privacy, adaptation of worksheets to be completed with blank paper, added verbal checks for understanding and attention, troubleshooting connection challenges, and a telehealth preparedness checklist (among other adaptations). In addition, local providers in Zambia reviewed the telehealth modifications and provided input that was incorporated into the final T-CETA manual. The CETA providers in Lusaka were provided additional training and supervision in T-CETA, including assessment of their foundational helping competencies using the Enhancing Assessment of Common Therapeutic factors Tool (ENACT) and related EQUIP resources (11).

Similar to CETA, T-CETA was developed for delivery by a range of providers, including nonprofessional providers without formal mental and behavioral health education or training. In this study, existing CETA providers were provided phones, data, and talk time to use for T- CETA sessions and weekly supervision. The T-CETA manual includes adaptations for video and telephone synchronous delivery, and both options were offered to all T-CETA clients. However, only telephone was feasible for all participants in this study. Each provider worked with their clients to set consistent weekly times for sessions, when the client was home and in a private and safe space. T-CETA clients received weekly, 1 h phone sessions. T-CETA providers were supervised *via* telephone by their respective supervisors 2 h each week.

### Qualitative Interviews

Semi-structured qualitative interviews were conducted with AYA following their T- CETA treatment *via* the telephone to maintain COVID-19 safety guidelines in December 2020. All interviews were conducted by the first author (SM), in either Bemba, Nyanja, or English, according to interviewee preference and primary language spoken. The interviewer ensured all AYA were in a private, confidential space. Interviews lasted approximately 30 min.

Interviews were not audio recorded due to privacy concerns *via* telephone and participant preference. Instead, the interviewer obtained permission from participants to keep detailed notes and record verbatim key quotes. No compensation was provided to participants. Interview questions aimed to explore AYA experiences and perceptions of receiving telephone-delivered CETA for mental health needs among young people in Zambia during the COVID-19 pandemic. Interview questions were designed to mitigate positive bias by including a balance of open- and close-ended questions that elicited both the strengths and challenges of telehealth CETA. For the full interview guide, see Supplementary Materials.

### Data Analysis

An inductive approach was used to collect and analyze the data, which allows for data- driven findings guided by study aims and research questions. Coding and analyses were conducted in Microsoft Excel. The first and second authors (SM, CF) created a final guidebook through the following processes: (1) review of all transcripts, (2) four interviews were randomly selected for consensus coding, (3) coders met to review codes and create initial codebook, (4) two additional interviews were coded according to preliminary codebook, and (5) coders reviewed codes and generated final codebook. The remaining interviews were split coded according to the final codebook. Reporting patterns were tabulated according to themes within the research aims, including advantages, barriers, and recommendations for telehealth delivery of CETA in Zambia.

Analyses were conducted in line with the thematic approach to explore response patterns that cross-cut interview questions and participants (17). Accordingly, analyses aimed to develop response-driven concepts that illuminate mental telehealth experiences in this population and context according to research aims. Broader sociocultural conditions that impact telehealth delivery and reception were integrated into the explanations and discussion of study aims.

### Data Trustworthiness and Triangulation

The full research team included both Zambian and American clinical researchers. Study design development, research aims, interview guide, implementation, translation, coding, member checking of results and manuscript preparation was completed collaboratively with Zambian and American team members, according to Community Based Participatory Action Research principles (18). The full team reviewed findings and provided input for final analyses and results.

## Results

Thematic analyses resulted in a range of subthemes across participants and the following research aim categories (1) Advantages of telehealth delivery of CETA, (2) Disadvantages or barriers to telehealth delivery of CETA, (3) AYA recommendations for optimizing telehealth CETA in Zambia. For themes, subthemes, and key AYA quotes, see Table 1.

**Table 1:** AYA reported themes, subthemes, and key quotes.

**Theme**	**Subtheme**	**Key participant quotes**
Advantages of T-CETA	Improvement in mental, behavioral, or social functioning	“I am beginning to feel less anxious, and I don't think about the incident as much as I used to in the beginning” “Talking to my counselor has made me feel better and things are a bit different for better” “I am feeling better than before though I feel I will eventually I will be ok” “The problem was somewhat dissolved, because I lost hope and the counselor constantly encouraged me not to lose hope. I am still under stress but not the way it was before counseling” “The problem was somewhat dissolved, because I lost hope and the counselor constantly encouraged me not to lose hope. I am still under stress but not the way it was before counseling” “I am able to find a way of been safe whenever I feel that my husband will start a fight when he is drunk”
	Feelings of comfort, engagement, and therapeutic rapport with provider	“I feel comfortable. The counselor talked to me about confidentiality, and he also told me to feel free” “I feel comfortable because the counselor encourages me to feel free and tells me about confidentiality” “I felt uncomfortable in the beginning and bored. After talking to her twice, I even wished she called me all the time. She was very encouraging” “I felt very comfortable, he was very encouraging, and we worked well together, and he was patient with me, we need to have more counselors in clinics use phones to call us for sessions”
		“I feel comfortable talking to my counselor apart from talking to her when my husband is home” “I feel comfortable but sometimes there are always people around me and I struggle to talk'
	Reduction of logistical barriers	“I feel comfortable using the phone especially now that it is raining, I don't have to go to the clinic” “I feel comfortable talking to my counselor on the phone because I don't have to go on a bus to meet with her”
Disadvantages or barriers to T-CETA	Difficulty finding a private space and time for T-CETA	“I feel comfortable talking to my counselor apart from talking to her when my husband is home” “I feel comfortable but sometimes there are always people around me, and I struggle to talk” “Talking to my counselor on phone is sometimes difficult because it is difficult to time the sessions. I may receive a phone call and that means my conversation with the counselor will be interrupted” “Sessions can be difficult during sessions, and I receive a call, that would interrupt my session with my counselor”
	Limited access to a phone or other device	“I feel comfortable but I suggest you buy me a phone because I have to use other people's phones to talk to my counselor”
	Challenges with reliable connectivity and access to electricity	“Yes, sometimes my phone cuts out”
AYA recommendations	Support finding private space	“I feel comfortable, apart from when I am in a bus or in front of people” “I feel comfortable talking to my counselor apart from talking to her when my husband is home” “I feel comfortable but sometimes there are always people around me and I struggle to talk”
	Support with phone provision	“I feel comfortable, but I suggest you buy me a phone because I have to use other people's phones to talk to my counselor”
	Include a face-to-face meeting	“I would like to meet face to face even though the phone therapy is helping” “Everything we discussed was good. I just wish I got to meet the counselor because she was very encouraging” “The phone counselor has been ok but I wish to meet in person”

### Advantages of Telehealth Delivery of CETA

#### Improvement in Mental, Behavioral, and Social Functioning

All T-CETA clients in this study participated in 5 to 7 sessions. Given the modular design of T-CETA, participants received a precision-based combination of treatment components depending on their presentation and comorbidities. Improvements in mental, behavioral, and social functioning were reported by AYA, including reductions in anxiety and posttraumatic stress due to strategies learned for changing unhelpful and “stuck” thoughts. AYA reported enhanced feelings of safety from interpersonal violence, noting the safety plans created with their CETA provider were instrumental in identifying and employing strategies for avoiding risky situations and activating key family members or friends for supporting the safety plan. For AYA in this study with problematic alcohol use, reductions in frequency and amount were reported, citing the effectiveness of utilizing alternative behavioral strategies when feeling an impulse to consume alcohol. Improved social relationships were reported with both family and friends, including less conflict and safer interactions with partners. Many AYA reported job loss due the pandemic and discussed the benefits of certain T-CETA elements to help with problem solving options to reduce COVID-related financial stressors, including finding work.

#### Feelings of Comfort, Engagement, and Therapeutic Rapport With Provider

AYA reported feelings of engagement and comfort with their provider, even with all interactions taking place *via* telephone. Specifically, AYA cited that having the provider introduce themselves *via* the phone, including their name, details of the study, their role on the study, sharing a common experience, and the reason why the AYA were reached *via* phone was critical to improving feelings of comfort. Overall, AYA reported confidence in the privacy of the conversations and the confidentiality of their health information and data. The T-CETA manual includes a thorough introduction session of what is included in CETA sessions with components that encourage participation, promote a sense of hope, and cover telehealth-specific considerations, including privacy and confidentiality *via* telephone, finding a safe space and secure connection, and in high-risk cases, strategies for maintaining safety (e.g., scheduling a session at a time the partner is not home; code words for if an abusive partner enters the room). In addition, AYA reported the punctuality and consistency of their T-CETA providers promoted a sense of comfort, trust, and engagement that facilitated strong therapeutic rapport.

#### Reduction of Logistical Barriers

AYA reported reduced logistical barriers, such as not having to travel to the clinic for sessions and the cost savings of telephone sessions relative to costs of traveling to a health care facility. In addition, AYA noted telehealth sessions were more time-efficient compared to the time for transport to and from a clinic, waiting for the provider, and waiting for an available space for sessions at the clinic when in-person. Telephone sessions also reduced other responsibility burden, including finding childcare. The majority of AYA noted increased perceptions of safety relative to in-person health services, due to the ability to maintain COVID- 19 safety guidelines and restrictions and not utilize public transportation. AYA reported holding misconceptions regarding COVID-19 infection and transmission (e.g., alcohol is a cure for COVID), and that maintaining access to a healthcare provider also allowed for the opportunity to gather trusted information on COVID infection, transmission, and safety measures. Further, AYA noted traveling to health care facilities can pose a safety risk if traveling at night, using public transportation, and walking long distances, all of which was eliminated by utilizing telephone-delivery of services.

### Disadvantages or Barriers to Telehealth Delivery of CETA

#### Difficulty Finding a Private Space and Time for T-CETA

Several AYA reported difficulties in access to a private and confidential space to participate in T-CETA sessions. AYA noted spaces available in their home were often proximal to others where the conversation could be heard. When the possibility of others hearing the conversation put the client at risk (e.g., in proximity of an abusive partner) sessions were postponed, which posed a challenge for session continuity and finishing treatment. Despite these challenges, AYA overall noted confidence in their confidentiality, due to the T-CETA introduction and provider-client problem solving other spaces and times for sessions. Other AYA reported that timing sessions with counselors was sometimes difficult and incoming calls would interrupt the session with the counselor. Several AYA noted their T-CETA providers attempted to problem solve barriers related to both the location and timing of T-CETA sessions but were faced with a limited array of options.

#### Limited Access to a Phone or Other Device

Some AYA in this study reported limited or inconsistent access to telephones or other communication devices to participate in T-CETA sessions, as most did not have personal phones. In this study, providers were given phones for T-CETA sessions, but did not provide phones for clients. AYA noted they needed to rely on recruiters, neighbors, or members of the household to have T-CETA sessions, which sometimes resulted in missed, shortened, or interrupted appointments. It is common in Zambian households for family members to share a single device, which impacts access and confidentiality for telehealth services.

#### Challenges With Reliable Connectivity and Access to Electricity

AYA reported difficulty maintaining a reliable connection for the duration of T-CETA sessions. This was occasionally due to poor service connectivity, and at other times due to a lack of electricity to keep devices charged. AYA noted mobile phone service providers and users in Zambia are often impacted by load-shedding, or planned electricity interruptions, that can impact both service connectivity and access to in-home electricity. Other AYA discussed inclement weather as another factor impacting reliable mobile phone services. Poor connectivity resulted in some missed, interrupted, and postponed T-CETA sessions.

### AYA Recommendations for Optimizing Telehealth CETA in Zambia

#### Support Finding Private Space and Time for Telehealth Sessions

When asked for recommendations on how to improve experiences with T-CETA, AYA noted needing additional support from providers in identifying private spaces for sessions. This included additional time spent with providers problem-solving available space in the home or in nearby settings. Identifying a private space for sessions is covered in the T-CETA introduction session, including problem-solving. However, AYA noted changes in their living situation during T-CETA treatment that required continued discussion and adjustment of the best space for sessions. Due to this, additional modifications were made to the T-CETA manual to discuss what space will be used for the upcoming session at the end of each call, and a check-in regarding space at the start of each session. Other AYA suggested identifying shared community spaces that could be reserved or designated for T-CETA services, such as in a church or nearby community center.

#### Support With Phone Provision

For AYA without consistent access to a telephone, it was recommended the CETA program provide phones or another mobile device. AYA noted a phone dedicated for telehealth services would promote consistency and adherence to T-CETA sessions, even if just made available for the duration of CETA treatment.

#### Include a Face-to-Face Meeting

Many AYA in this study reported a preference for at least one face-to-face meeting with their counselor to increase comfort, familiarity, and rapport building. Although AYA generally felt comfortable working with their provider *via* telephone, some noted it took longer than with an in-person provider and suggested an initial in-person meeting or quick meet-and-greet prior to starting T-CETA to increase comfort. Some AYA described a preference for a hybrid model of CETA, in which some sessions are held in-person and others *via* telephone, depending on availability, session content, and preference.

## Discussion

This qualitative study explored AYA experiences with a telehealth-delivered evidence- based mental and behavioral health program, T-CETA, during the COVID-19 pandemic in Lusaka, Zambia. Overall, AYA described T-CETA as a feasible, acceptable, and effective program for mental and behavioral health treatment in Zambia. AYA reported confidence in their providers maintaining client privacy and confidentiality. Of note, the T-CETA treatment manual includes best practice telehealth-specific discussion of privacy and confidentiality, such as ensuring the client is in a private space, others cannot hear the conversation, and clearly describing what information is being recorded during the session and where notes are stored. Additional modifications were included to check-in with clients each session regarding space, privacy, and confidentiality, both in the current session and planning for the next session.

Although AYA reported high levels of stress and anxiety during the pandemic due to isolation, job loss and other financial strain, they described positive clinical outcomes following their participation in T-CETA. This supports other study findings of respondents stating their telehealth experience was “just as good as” or “better than” their traditional in-person medical care (19). In both this study and elsewhere, respondents perceive telehealth as an acceptable, viable, and useful modality for healthcare appointments even after the COVID-19 pandemic ends (19). The T-CETA manual includes elements specific to managing daily life stressors, improving interpersonal relationships, and provides coping and problem-solving skills to improve certain financial situations. AYA noted CETA skills helped them secure jobs and reduce financial stress. Further, AYA noted they worked with T-CETA counselors to problem solve ways to reduce feelings of isolation *via* safe social activities and communication strategies. These findings suggest these skills can be effectively taught remotely to improve daily functioning for AYA in Zambia. Overall, these findings align with a growing body of literature supporting the use of telehealth care delivery (20), with less cost for both patients and clinicians compared to traditional visits (20).

Emergent research from other countries has identified similar advantages to remote care, such as increased accessibility and convenience for those facing geographical barriers as well as convenience and communication within and between mental health teams (21, 22). Telehealth services also saved client costs, eliminating the need for transport fees, and was in-line with COVID-19 safety guidelines. Transport in Zambia is particularly risky for COVID transmission, given the primary form of transportation is minibuses that are overcrowded and maintain minimal enforcement of COVID safety recommendations, such as masking. Overall AYA experiences with T-CETA, including reported improvements in social, emotional, and behavioral functioning, suggest non-professional providers can be trained to deliver tele-mental health treatments in ways feasible and acceptable to recipients of care in Zambia.

Disadvantages were also noted by AYA in this study, including difficulty accessing reliable devices and private spaces to participate in sessions, which left unaddressed, are a limitation to consistent access to telehealth mental health services in Zambia. Although telehealth reduced travel barriers to a healthcare facility, AYA in this study reported challenges with securing a private space for T-CETA sessions in their homes or nearby settings. In addition, technology disruptions, such as unreliable phone network connectivity and inconsistent access to a device, hindered access to T-CETA sessions. The cost of obtaining a mobile device and talk time/data was reportedly prohibitive for some AYA in this study, which aligns with other studies in global settings during COVID-19 (23). In addition, load-shedding, or planned electricity interruptions, are common in Zambia and resulted in some sessions being canceled. These findings align with research suggesting the use of a “hub and spoke” model, in which facilities in conveniently located rural or community sites are designated access points to a device, a strong connection, and a private and confidential space (spokes) that connect clients with the trained T- CETA providers at a healthcare facility or other site (hub) (24). In line with AYA recommendations, churches may serve as an appropriate and conveniently located site for telehealth access points in Zambia. Large spaces such as churches could also offer safe, physical distancing while supporting a convenient space and a supported network connection in the context of pandemics such as COVID-19.

Other recommendations from AYA included ensuring at least one face-to-face meeting with their provider to improve engagement and familiarity. Telehealth recipients in other contexts, including high-income countries, have reported the same preference, including a desire for hybrid models of care that include a combination of in-person visits, video visits, and telehealth sessions (25). Additional adaptations to the T-CETA manual, and other telehealth manuals, are merited to include an option for the initial meeting to occur in-person or *via* video call, with problem solving barriers to those modalities.

AYA also noted the need for additional support in securing mobile devices and finding private space for sessions. To facilitate private spaces, future implementation can include the following precautions: 1. Guide patients to inform family members or others in the household in advance that a session will be taking place, 2. Facilitating a pre-session check-in to discuss where the session will be conducted and to problem-solve alternative

locations, 3. Encourage the use of headphones during sessions, and 4. Consider outdoor spaces to participate in sessions, if private. Additional planning prior to conducting sessions for patients can enhance privacy and maintaining confidentiality.

Overall, findings point to telehealth as an important strategy to allow mental and behavioral health services to continue during public health crises such as COVID-19. Findings suggest tele-mental health should be integrated into the existing national strategic plan for Zambia to increase access to evidence-based telemedicine modalities, particularly in rural and other hard-to-reach populations (26). Given overall positive experiences with telehealth, findings suggest the benefits in continuing with the T-CETA for future service delivery even as pandemic restrictions ease.

## Limitations And Future Directions

Responses elicited during these qualitative interviews are susceptible to social desirability bias given participants were recipients of free care and were interviewed by someone more senior, a phenomenon particularly applicable to the Zambian context. Future studies of telehealth acceptability should include other telehealth delivery modalities (such as a video call for initial introduction session or all sessions), multi-method data collection, including anonymous self-report, focus groups discussions, and interviews with a trained peer to increase comfort with open conversation. The input of T-CETA providers and their experiences delivering telehealth would be useful in advancing telehealth feasibility with providers and additional adaptations to ease provider burden. In addition, future studies should include data collection points at various times throughout telehealth treatment to capture those who may not complete treatment due to internal or external barriers with telehealth. Given AYA reports of difficulty purchasing mobile devices for consistent access to their telehealth providers, future studies should evaluate the cost- effectiveness of providing devices and talk time to patients. This may include dedicated devices maintained by the facility or program that are lent to clients only for the duration of the CETA treatment. Providing mobile devices may limit scalability and sustainability in some low resource settings, however, cost savings from eliminated transport costs and other logistical barriers to attending sessions (childcare provision, loss time at work, etc.), particularly in rural settings, is likely to result in overall greater cost-effectiveness for telehealth delivery even with the provision of temporary use phones.

## Conclusion

Overall, AYA reported T-CETA services as acceptable and clinically useful, with some logistic and sociocultural recommendations to improve consistency and familiarity with telehealth providers. Additionally, experiences reported by AYA indicate willingness to engage in telehealth mental and behavioral health services during public health crises. Findings support the continued scaling of telehealth services in Zambia and other LMIC to increase consistent access to evidence-based care.

## Data availability statement

The raw data supporting the conclusions of this article will be made available by the authors, without undue reservation.

## Ethics statement

Ethical approval for this study was granted by the University of Zambia Biomedical and Research Ethics Committee (UNZABREC) approval number—ref-008-02-19 and the Johns Hopkins School of Public Health (JHSPH) Institutional Review Board Office, approval number—IRB No. 00009259. Written informed consent was obtained for all participants. For adolescents younger than 18 years, written informed assent and parental permission was obtained.

## Author contributions

SM-M: writing initial draft, data analysis, review and editing, and project manager. CF: writing initial draft, project design, data analysis, and manuscript review and editing. KM: project design, project oversight, manuscript review and editing, clinical supervision, and co-investigator. JK: project design, manuscript review and editing, and data analysis. SS: project design, manuscript review and editing, and clinical supervision. MM: project design, manuscript review and editing, and project management. BK: project design, manuscript review and editing, data analysis, EQUIP investigator, and final review before submission. GP: manuscript review and editing, data analysis, and EQUIP investigator. IS: project design, manuscript review and editing, and principle investigator. LM: project design, manuscript review and editing, and principle investigator. All authors contributed to the article and approved the submitted version.

## Funding

Funding for the WHO EQUIP initiative is provided by USAID.

## Conflict of Interest

The authors declare that the research was conducted in the absence of any commercial or financial relationships that could be construed as a potential conflict of interest.

## Publisher's Note

All claims expressed in this article are solely those of the authors and do not necessarily represent those of their affiliated organizations, or those of the publisher, the editors and the reviewers. Any product that may be evaluated in this article, or claim that may be made by its manufacturer, is not guaranteed or endorsed by the publisher.
