# Supplementary material for: Experiences and Perceptions of Telephone-delivery of the Common Elements Treatment Approach for Mental Health Needs Among Young People in Zambia During the COVID-19 Pandemic
Source: Front Public Health. 2022 Oct 13;10:906509. doi: 10.3389/fpubh.2022.906509 (PMC9610836; doi:10.3389/fpubh.2022.906509)
Supplement: Supplementary file 1 [file Data_Sheet_1.pdf]

## Qualitative Guide for CETA clients after completing TCETA

1. What was the problem(s) you were seeking/getting help for?

2. What did you do talk about with the phone counselor that was helpful? What part of the phone call(s) has been *most* helpful?

3. Have you ever sought care for this problem before? If so, how was your experience with counselor the same or different from the help you received before?

4. How are your problem(s) going compared to before you talked with the phone counselor?

5. How has the treatment affected the way you deal with your problem(s)?

6. Tell me about how you felt talking with the phone counselor – (e.g., comfortable, uncomfortable, safe/unsafe, excited, bored…etc.)

7. How could you tell if the phone counselor cared about you and your problem(s)?

8. What were things that you would have liked the phone counselor to do differently?

9. What were difficulties or challenges with the phone call(s) if any?
